# Membrane Interactions of Latarcins: Antimicrobial Peptides from Spider Venom

**DOI:** 10.3390/ijms221810156

**Published:** 2021-09-21

**Authors:** Parvesh Wadhwani, Saiguru Sekaran, Erik Strandberg, Jochen Bürck, Archana Chugh, Anne S. Ulrich

**Affiliations:** 1 Institute of Biological Interfaces (IBG-2), Karlsruhe Institute of Technology (KIT), P.O. Box 3640, 76021 Karlsruhe, Germany; parvesh.wadhwani@kit.edu (P.W.); erik.strandberg@kit.edu (E.S.); jochen.buerck@kit.edu (J.B.); 2 Kusuma School of Biological Sciences, Indian Institute of Technology Delhi, Hauz Khas, Delhi 110016, India; saiguru479@hotmail.com (S.S.); archana.chugh@bioschool.iitd.ac.in (A.C.); 3 Institute of Organic Chemistry, Karlsruhe Institute of Technology (KIT), Fritz-Haber-Weg 6, 76131 Karlsruhe, Germany

**Keywords:** spider venom latarcin peptides, membrane permeabilization, solid-state ^15^N- and ^31^P-NMR, oriented circular dichroism, fluorescence vesicle leakage, antimicrobial, hemolysis

## Abstract

A group of seven peptides from spider venom with diverse sequences constitute the latarcin family. They have been described as membrane-active antibiotics, but their lipid interactions have not yet been addressed. Using circular dichroism and solid-state ^15^N-NMR, we systematically characterized and compared the conformation and helix alignment of all seven peptides in their membrane-bound state. These structural results could be correlated with activity assays (antimicrobial, hemolysis, fluorescence vesicle leakage). Functional synergy was not observed amongst any of the latarcins. In the presence of lipids, all peptides fold into amphiphilic α-helices as expected, the helices being either surface-bound or tilted in the bilayer. The most tilted peptide, Ltc2a, possesses a novel kind of amphiphilic profile with a coiled-coil-like hydrophobic strip and is the most aggressive of all. It indiscriminately permeabilizes natural membranes (antimicrobial, hemolysis) as well as artificial lipid bilayers through the segregation of anionic lipids and possibly enhanced motional averaging. Ltc1, Ltc3a, Ltc4a, and Ltc5a are efficient and selective in killing bacteria but without causing significant bilayer disturbance. They act rather slowly or may even translocate towards intracellular targets, suggesting more subtle lipid interactions. Ltc6a and Ltc7, finally, do not show much antimicrobial action but can nonetheless perturb model bilayers.

## 1. Introduction

All species constitutively produce antimicrobial peptides (AMPs) to defend the host from undesired microbial invasion [1]. Venoms, on the other hand, are toxic and complex secretions applied through the sting, teeth, fangs, or harpoons of venomous animals, such as insects, snakes, cone snails, and spiders [2]. The primary use of venom is to fight aggressors and deter predators, in addition to paralyzing and/or killing and preserving prey. Interestingly, depending on the organism’s habitat, many venoms are evolutionarily optimized to also contain broad-spectrum antimicrobial peptides. Most of these short, amphiphilic, and often cationic AMPs tend to act on bacterial membranes and hence show an intrinsic tendency to destabilize the lipid bilayer. This property of AMPs has been exploited since their discovery in the 1990s, with the aim of optimizing their potential against microbial infections, evaluating their cytotoxicity toward cancer cells, and reducing their hemolytic effects against erythrocytes, amongst other potential applications. AMPs are essential components of the venom of many (but not all) spiders. From an estimated 150,000 species of spiders, only a quarter (approximately 43,000) of spider species are cataloged, and the venom components of only 174 spiders have been described [3,4,5]. These data clearly indicate the dearth of information available on potentially promising molecules that are currently lacking attention and waiting to be optimized for a given application. By some estimate, spider venom has been predicted to contain over 10 million bioactive peptides [6] with a wide range of activities, including analgesic, antiparasitic, cytolytic, antitumor, and anti-inflammatory activities. However, only a minuscule fraction of these peptides have been studied or biophysically characterized to be exploited for human benefit.

In this work, we focus on the membrane interactions of one such group of seven linear helical peptides called latarcins (Table 1). Latarcins are the C-terminal fragments of larger precursor proteins isolated from the venom of the spider *Lachesana tarabaevi*. They consist of a signal peptide, followed by an acidic fragment that is cleaved off post-translationally, resulting in one common group of seven AMPs. They were discovered, isolated and characterized by Kozlov et al. in 2006 [7]. Latarcins were isolated from crude venom by HPLC fractionation, characterized by mass spectrometry, and sequenced using amino acid analysis. Table 1 shows the primary structure of latarcins. It was found that Ltc3a, Ltc4a, and Ltc5a are amidated; the other four latarcins bear a free -COOH group at the C-terminus. Once the sequence identity had been established, the peptides were chemically synthesized. As with the majority of AMPs, latarcins were found to be unstructured in water, whereas, in 50% trifluoroethanol (TFE), they are able to fold into α-helices [7]. It is well known that TFE promotes α-helical structures [8,9] and that a majority of membrane-active peptides tend to fold into helices in this solvent system. These helices, however, may or may not represent the state of the peptide when it interacts with the target biomembrane. 

Latarcins have been demonstrated to exhibit high antimicrobial, cytolytic, and membrane perturbing activity, with the exception of Ltc6a and Ltc7 [7]. While the exact role of these multifunctional peptides in the spider venom remains unclear, it appears that their major contribution is toward the paralyzing and cytolytic effects [7]. Furthermore, based on the observed synergistic effects of the venom components with other neurotoxins, it is also proposed that latarcins may facilitate the passage of neurotoxins through cellular barriers and sterilize the prey, in addition to protecting the venom glands from external microbial infections [10]. Most investigations in the past have restricted their focus to Ltc1 [11] or Ltc2a [12]. Based on circular dichroism spectroscopy (CD) and solution NMR data in SDS micelles and restrained Monte Carlo simulations, a membrane-bound structure has been presented for these two selected peptides. In an attempt to fine-tune the antimicrobial property of Ltc2a, it was further shown that the hydrophobic N-terminal part in Ltc2a triggers hemolytic side effects [13]. The same has not been shown for other latarcins. Although *L. tarabaevi* is reported to be a rich source of linear membrane-active compounds [14], i.e., latarcins and other peptides, there has been no study thus far in which all members of the latarcin family have been systematically studied in the presence of membranes. Comparative studies on all members of the latarcin family include only a determination of their antimicrobial and hemolytic activities [7,10]. Biophysical studies have only been performed on Ltc1 and Ltc2a [7,11,12]. 

**Figure 1:**
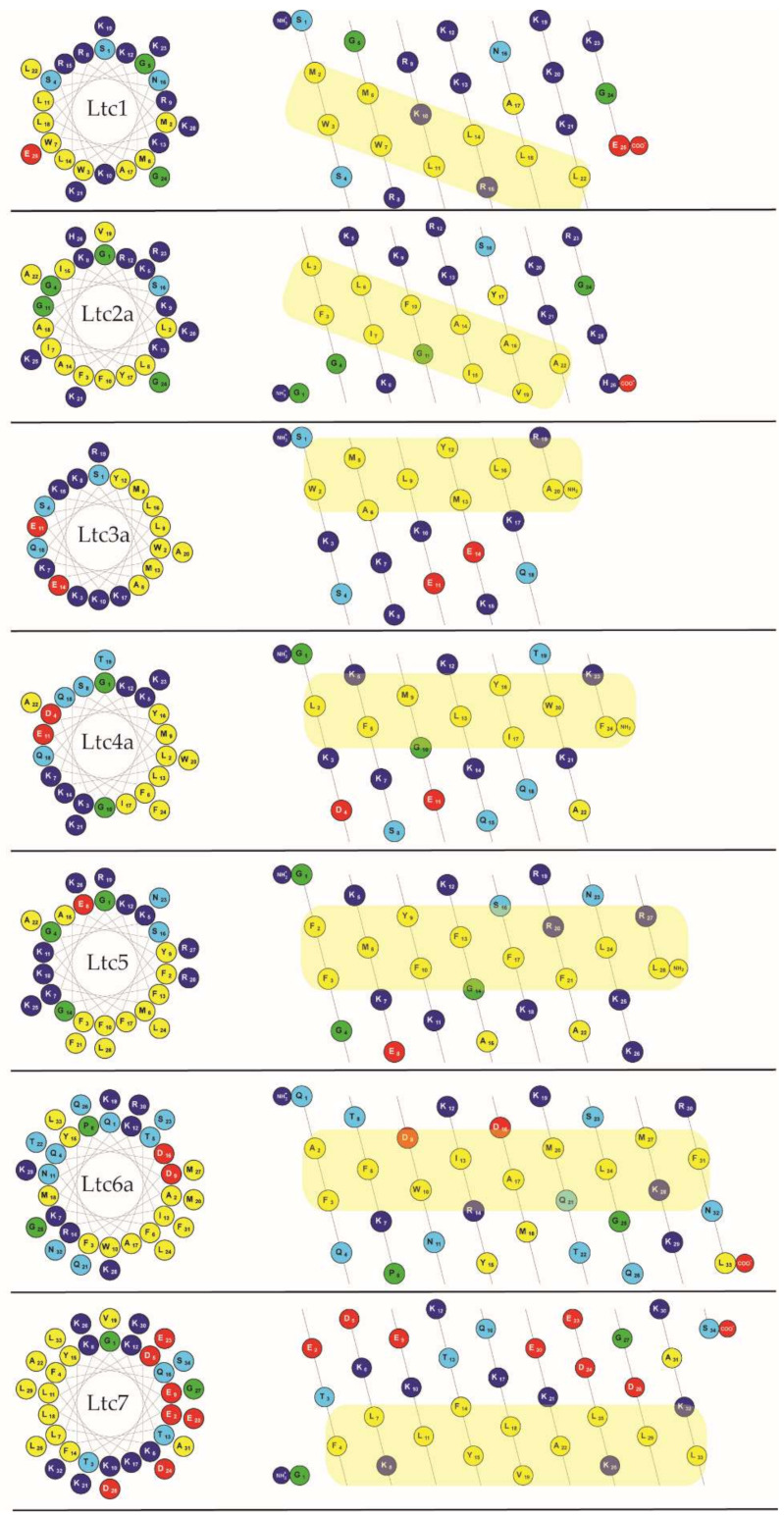
Helical wheels and helical meshes of the amphiphilic latarcin peptides (prepared using the Protein Origami web application found at http://www.ibg.kit.edu/protein_origami/ [15]). It is assumed that the peptides are fully helical (see text), and the hydrophobic face has been highlighted in yellow. Residues are color-coded: yellow—hydrophobic; light blue—polar; dark blue—cationic; red—anionic; green—Gly/Pro.

All latarcins are positively charged (largely Lys rather than Arg) and contain several aromatic amino acids (Table 1), but they do not show any sequence homology. Figure 1 (left column) illustrates how all latarcins can form amphipathic structures when folded as an α-helix, as the hydrophobic residues are segregated on one face of the helical wheel and the charged (and polar) ones on the opposite face. This property implies that they should all bind favorably to lipid membranes in this conformation. However, there are some noticeable differences among this group of peptides. For example, the net charge varies from +2 to +10, and when considering the peptide lengths between 20 to 34 amino acids, the average net charge per residue varies from +0.06 to +0.40. According to the sequences and other properties listed in Table 1 as well as the patterns shown in Figure 1, we propose here and will proceed to group the latarcins into four types with related structural properties:

Group A peptides (Ltc1 and Ltc2a) consist of 25–26 residues and have a free C-terminus. They carry the highest net charge, resulting from 9 to 10 exclusively cationic groups without any anionic residues, besides 9–11 hydrophobic amino acids. In the helical mesh representation, it can be seen (better than in the helical wheel) that the strip of hydrophobic residues runs diagonally along/around the helix axis. Notably, in Ltc1, the hydrophobic strip is disrupted by two cationic residues (Lys10; Arg15), which is not the case in Ltc2a. 

Group B peptides (Ltc3a and Ltc4a) are the shortest peptides with 20-24 residues and possess an amidated C-terminus. Their net charge of +6 results from 8 positively and 2 negatively charged groups, besides 8–9 hydrophobic amino acids. They display a typical amphiphilic profile, with a straight strip of hydrophobic residues that are flanked by cationic residues (nicely seen in the helical wheel representation).

Group C comprises only a single peptide (Ltc5) with 28 residues and an amidated C-terminus. It has a net charge of +10 and an unusually high number of 7 aromatic amino acids (mainly Phe) amongst 12 hydrophobic ones. The helical mesh representation indicates that the hydrophobic strip becomes less defined towards the C-terminus.

Group D (Ltc6a and Ltc7) are the longest peptides with 33–34 residues, possessing a free C-terminus. Ltc6a is rich in polar residues, while Ltc7 is particularly rich in charged residues (8 Glu/Asp and 10 Lys). The resulting net charge of +5 and +2, respectively, is relatively low. Some 12–13 hydrophobic residues contribute to a long and well-defined amphiphilic profile. 

Given the similar properties, we expect that the peptides within each group might also display similar activities in the presence of membranes. For example, one can directly predict from the charge that peptides in Groups A and C (Ltc1, Ltc2a and Ltc5), which have the highest charge per residue of 0.36, should be favorably attracted to bacterial membranes that are generally anionic. Previous studies have shown some indication in this direction. In contrast, Group D peptides (Ltc6a and Ltc7), which have a lower net charge, are expected to be less active against bacteria, while Group B should be moderately active. From the helical wheels and mesh representations shown in Figure 1, one can also predict that all latarcins should be able to fold into amphipathic helices and thereby interact with lipid membranes. Previous studies have only shown a helical fold in TFE, and there are no data on the entire set of latarcins to demonstrate their folding into helices upon binding to a membrane. With the current understanding of the physical properties of peptides, it is not possible, however, to predict how such helical peptides will behave when bound to a membrane, i.e., whether they will stay on the surface, or become tilted, or insert as oligomers, or have any tendency to aggregate non-productively. It is also not possible to predict whether they would form any transient or stable pores or cause any damage to the lipid bilayer that can be assessed by monitoring vesicle leakage. 

Therefore, for the first time, we have performed a combined study of the full set of all seven latarcins using several biophysical techniques and biological assays. In particular, we have characterized the structure of these antimicrobial peptides in lipid bilayers using CD, oriented CD (OCD), and solid-state ^15^N-NMR methods to assess the predicted and previously proposed structures [10,11,12] of the peptides per se and in the presence of lipid bilayers. Using solution CD, one can determine whether they are indeed helical in the presence of lipids, as implied in Figure 1 and demonstrated in TFE [7]. If they are helical, it is important to know how they integrate into the membrane. This information can provide important clues on the mechanism of action of these peptides on the target cell, i.e., whether they stay folded or rather aggregate, or assemble to form pores in the bilayer, or have any other targets inside the bacterial cell. For this purpose, we used OCD and solid-state ^15^N-NMR to determine the helix alignment in oriented membrane samples. Furthermore, we determined the antimicrobial and hemolytic properties of all peptides and performed vesicle leakage to assess their intrinsic toxicity toward model membranes of a given composition. 

## 2. Results

### 2.1. Peptide Synthesis

All peptides were successfully prepared using solid-phase synthesis and purified before use. To avoid purification troubles, a low load (LL) Wang resin (0.3 mmol/g) was used for the longest latarcins (Ltc6a and Ltc7). This method resulted in crude peptides containing a higher amount of the desired product as the major product. Other latarcins were synthesized either on a Wang resin with normal loading (0.5–0.8 mmol/g) or on a rink amide MBHA resin (0.78 mmol/g), depending on whether the C-terminus was an acid or an amide. In each latarcin peptide, a single ^15^N-NMR label was introduced in the backbone amide at a residue in the middle of the sequence (residue underlined in Table 1). Analytical chromatograms of the purified peptides are shown in the Supporting Information (Figure S1).

### 2.2. Circular Dichroism Spectroscopy

**Figure 2:**
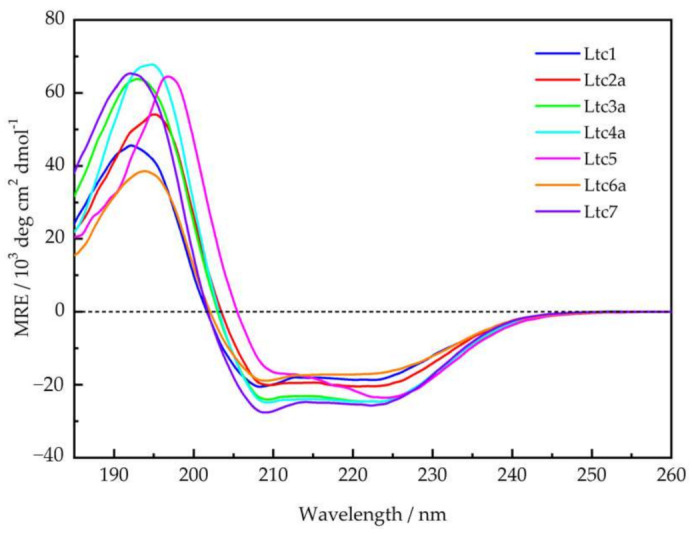
Circular dichroism spectra of latarcins in the presence of small unilamellar vesicles made from DMPC/DMPG (7/3) measured at a molar peptide-to-lipid ratio (P/L) of 1/50 at 30 °C.

Most membrane-active peptides tend to be unstructured in aqueous solution, i.e., in the absence of lipids or a membrane-mimicking environment, and all latarcins were reported to be unstructured in water [7]. As a reference, we checked their secondary structure using solution CD in 10 mM phosphate buffer at a defined pH of 7 and found that all peptides are indeed largely unfolded under these conditions (Figure S2). Ltc3a shows a noticeable helical fold (ca. 30%) under these conditions. Next, we compared the secondary structure of the full set of latarcins in the presence of lipid membranes, for which there are no complete data available yet. Figure 2 shows that in the presence of small unilamellar vesicles composed of DMPC/DMPG (7/3), all latarcins fold into α-helices at a molar peptide-to-lipid ratio (P/L) of 1/50, as expected. A pronounced helicity had been reported in helix-promoting solvents, such as TFE/H_2_O (50/50 *v*/*v*) [7], with a positive band centered at 191 nm—with the exception of Ltc5, which had a positive band at 195 nm. In TFE, the two characteristic negative bands of an α-helix center at 208 nm and 222–223 nm were observed for all latarcins. In our lipid mixtures, however, several peptides show significant deviation from the idealized α-helix observed in TFE. While there is a relatively small variation in the spread of the negative band intensities among the latarcins, there is a noticeable shift in the positive maxima between the different peptides, as seen in Figure 2. Ltc1, Ltc3a, and Ltc7 show a positive band around 192-193 nm, while for Ltc4a and Ltc6a, the band is slightly shifted to 194 nm. Ltc2a shows a broad positive band at this wavelength, and Ltc5 displays the most prominent red-shift of the positive band with a maximum of 197 nm. The negative bands in Ltc5 are also shifted toward longer wavelengths. The change from an unordered structure or nascent helical conformation in the absence of lipids (Supplementary Figure S2) to a largely α-helical folded structure in the presence of lipids indicates that latarcins have a high tendency to bind to lipids and undergo a structural transition in response to their environment. 

### 2.3. Minimum Inhibitory Concentration 

**Figure 3:**
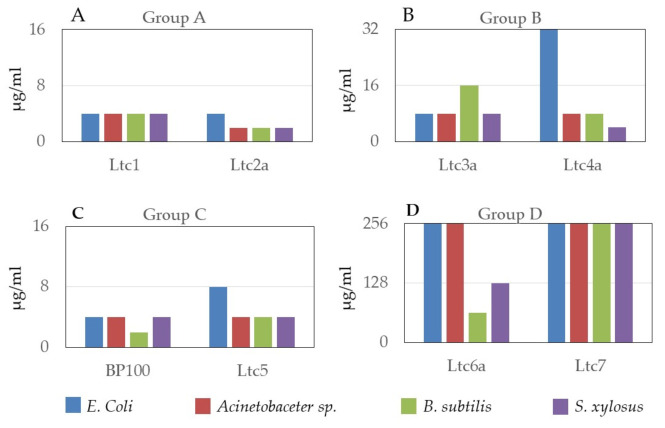
Minimal inhibitory concentration (MIC) values of all latarcins determined for two Gram-positive (*Bacillus subtilis* subsp. *spizizenii* and *Staphylococcus xylosus)* and two Gram-negative strains (*Escherichia coli* and *Acinetobacter* sp.). (**A**) Group A peptides (Ltc1 and Ltc2a); (**B**) Group B peptides (Ltc3a and Ltc4a); (**C**) Group C peptide (Ltc5) and positive control (BP100); (**D**) Group D peptides (Ltc6a and Ltc7).

The effect of latarcins against four types of bacteria was determined using a minimum inhibitory concentration (MIC) assay on two Gram-positive strains (*Bacillus subtilis* subsp. *spizizenii* and *Staphylococcus xylosus*) and two Gram-negative strains (*Escherichia coli* and *Acinetobacter* sp.). These results are shown in Figure 3. Although the antimicrobial activity of latarcins is well documented, these experiments served to ensure that all studies were performed on molecules that show the expected antimicrobial activity. Our results confirm that among latarcins, Ltc1, Ltc2a, and Ltc5 are the most active against the strains used in this study, as expected from their charge/residue ratio (Table 1) and from their large hydrophobic sector seen in Figure 1. Ltc3a and Ltc4a are slightly less active than Ltc1, Ltc2a, and Ltc5. Ltc6a is only active toward Gram-positive strains, whereas Ltc7 can be considered inactive, as it did not inhibit bacterial growth even at the maximum peptide concentration of 256 µg/mL used here.

### 2.4. Hemolytic Activity 

**Figure 4:**
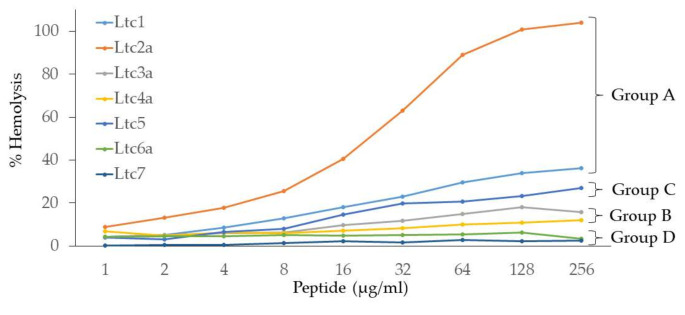
Hemolytic activity of latarcins as a function of the peptide concentration.

Compared to other antimicrobial membrane-active peptides, latarcins show rather low hemolytic activity, which makes this set of peptides interesting for further pharmacological optimization. A hemolysis assay was performed to determine the activity of latarcins against human red blood cells, allowing us to compare the results to those of previous experiments in which rabbit erythrocytes were used [7]. The activity was determined for each latarcin as a function of increasing concentration, up to a maximum of 256 µg of peptides per mL. The hemolytic activity of latarcins is seen to vary considerably among the different peptides. Figure 4 shows that Ltc2a exhibits 100% hemolysis at 128 µg/mL. The percentage hemolysis of the other peptides was determined at the highest tested concentration of 256 µg/mL. Our results show that Ltc1 resulted in 36% hemolysis, followed by Ltc5, which resulted in 27%. Ltc3a and Ltc4a showed low hemolysis of 16% and 12%, respectively, at 256 µg/mL. Ltc6a and Ltc7 had the lowest hemolytic activity (<3%). These hemolysis data demonstrate that peptides in any group show comparable activity, with the exception of Ltc 2a. Furthermore, our results show that the peptides with higher antimicrobial activity also display higher hemolytic activity, suggesting that this effect is due to an intrinsic perturbation on the lipid bilayer. 

### 2.5. Vesicle Leakage Activity 

**Figure 5:**
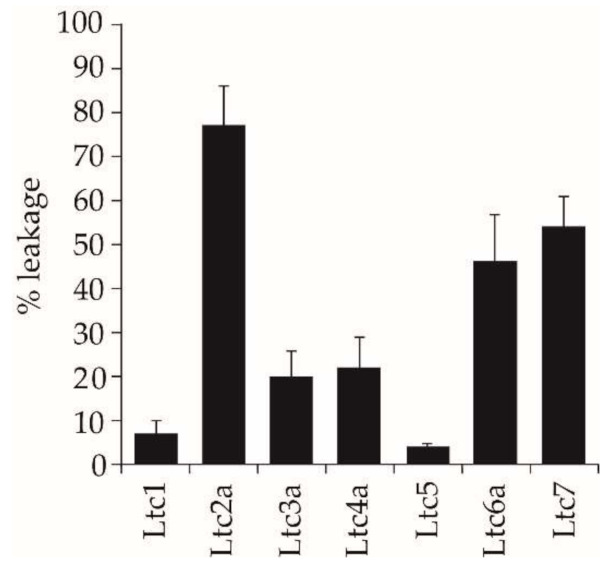
Peptide-induced leakage of ANTS/DPX in large unilamellar vesicles made of POPC/POPG (1/1) lipids at a P/L of 1/50.

The ability of the peptides to induce leakage in large unilamellar vesicles (≈120 nm diameter) of POPC/POPG (1/1) was studied using a fluorescence assay using ANTS/DPX, as described previously [16,17,18]. The different latarcins resulted in very different extents of leakage, as seen in Figure 5. The highly membrane-active peptide Ltc2a (a strongly antimicrobial and hemolytic agent) caused the most vesicle leakage (75%). Unexpectedly, Ltc6a and Ltc7, which had shown a very low antimicrobial and hemolytic activity, were found to give a high degree of 50% leakage. Ltc3a and Ltc4a induced 20% leakage, while Ltc1 and Ltc5 caused less than 8%. These data clearly demonstrate that the action of each peptide is highly dependent on the composition of the target lipid membrane.

To find out whether the latarcins display any synergistic effects amongst each other, as has been reported for other classes of antimicrobial peptides, further leakage experiments were performed with pairwise combinations. Each peptide was studied at P/L = 1/100 alone, as well as in combination with the other six peptides, where each peptide was present at P/L = 1/100. The extent of leakage for every combination was compared to the sum of the individual leakages. For synergy to be confirmed, the synergy factor needs to be at least 2. We found, however, that for all peptide combinations, the synergy factor was close to or below 1 (Figure S3). It can thus be concluded in the leakage assay that there is no synergy amongst any pair of latarcins.

### 2.6. Oriented Circular Dichroism

While CD is a quick and easy method to determine the secondary structure of any peptides when bound to a membrane, oriented CD (OCD) can be used to estimate the orientation of an α-helical peptide with respect to the membrane normal [19,20]. From Figure 2, we know that latarcins are helical, so we can now use OCD to distinguish whether these helices are bound flat on the bilayer surface, tilted obliquely into the membrane, or inserted fully in an upright orientation in the bilayer. 

**Figure 6:**
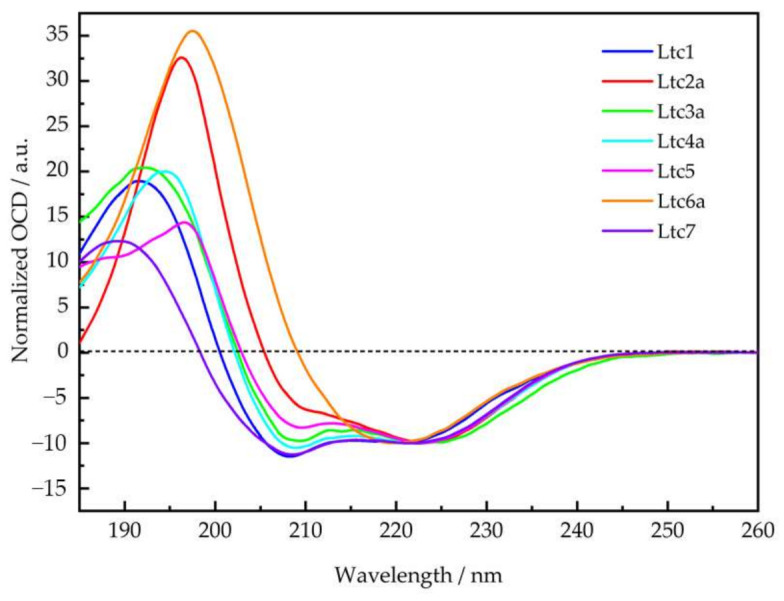
Oriented CD spectra of latarcins in oriented membrane composed of DMPC/DMPG (7/3) at a P/L = 1/100 measured at 30 °C.

Figure 6 shows the OCD data of all latarcins measured in DMPC/DMPG (7/3) at a P/L of 1/100. This is the same lipid composition and peptide-to-lipid ratio as was used in conventional CD above (see Figure 2), but in contrast to the vesicular samples containing excess water, the oriented membranes for OCD are just about saturated with water. Our OCD results confirm that all peptides are α-helical in macroscopically oriented membrane samples, with the exception of Ltc6a. Notably, in the presence of excessively hydrated vesicles, Ltc6a also had the lowest helical fold according to conventional CD (Figure 2, Table S1). Here, in the absence of bulk water (Figure 6), it actually displays characteristic spectral features of a β-pleated conformation with a broad and intense positive band centered around 197 nm and a less intense negative band at 219 nm.

The sign and intensity of the 208 nm band provide a characteristic signature of the helix tilt angle in the membrane [19]. For a helical peptide that lies flat on the bilayer surface, this band is the most intense. When the helix is obliquely tilted in the membrane, the 208 nm band intensity diminishes, as seen in the spectrum of Ltc2a. Our data show that Ltc2a is, in fact, the most tilted peptide among all latarcins. Ltc5 also appears to be tilted in the membrane according to the reduced intensity of the 208 nm band, but we note that it shows absorption flattening towards the low wavelength region, which is indicative of peptide-peptide interactions. That is, a similar reduction in the intensity of the 208 nm band was also seen in the solution CD of Ltc5. We, therefore, believe that this may be due to differential scattering artifacts in the OCD spectrum caused by vesicle clustering, possibly due to the presence of a large number of aromatic residues in Ltc5 and that the peptide may, in fact, be surface-bound. Ltc3a and Ltc4a seem to show only a modest tilt or possibly an averaged spectrum of two states that are fluctuating between surface-bound and tilted, according to the intensity at 208 nm. The spectra of Ltc1 and Ltc7, due to their pronounced negative signals at 208 nm, indicate that both these peptides remain flat on the membrane surface under the conditions of OCD. 

### 2.7. Solid-State ^15^N-NMR and ^31^P-NMR

**Figure 7:**
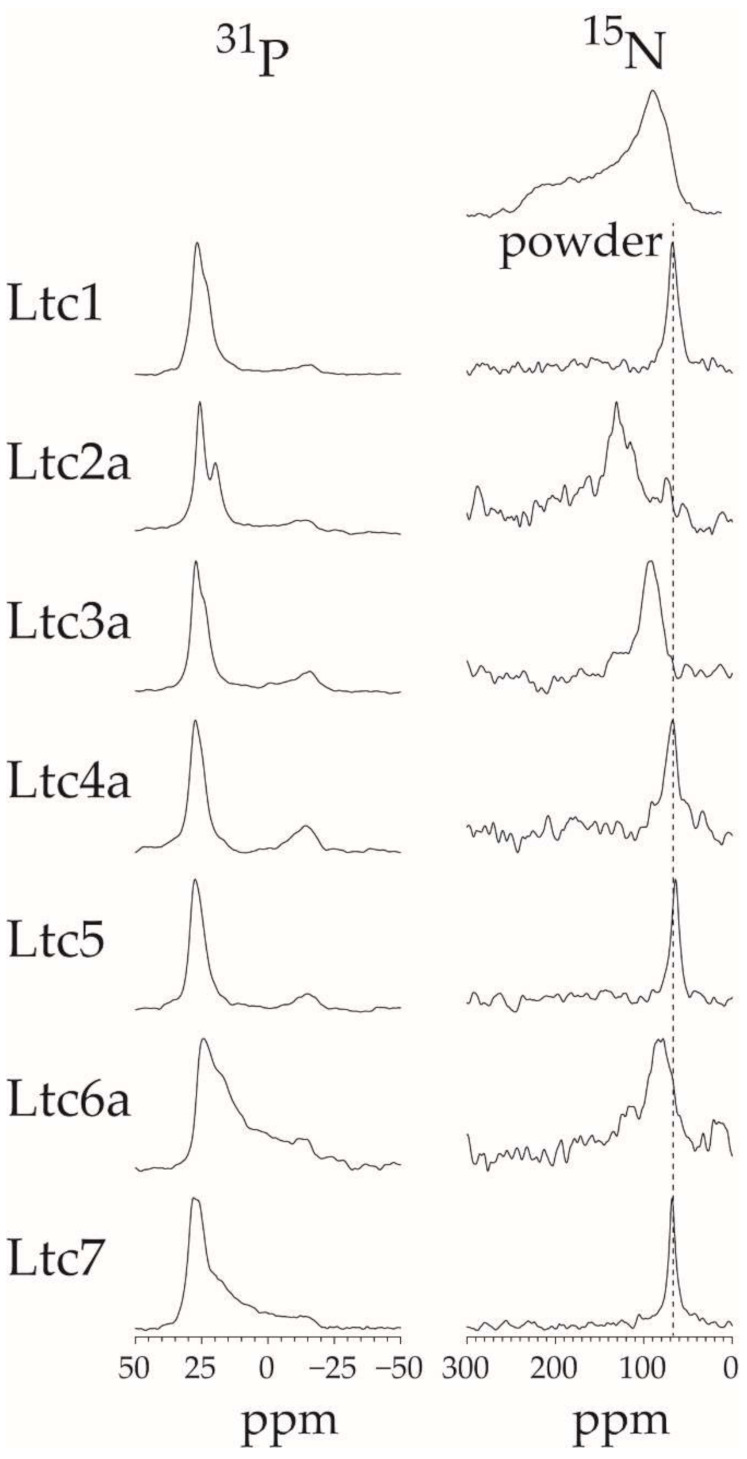
Solid-state NMR of ^15^N-labeled latarcin peptides in oriented samples of DMPC/DMPG (7/3), showing ^31^P-NMR of the phospholipids on the left and ^15^N-NMR of the peptides on the right. The ^31^P peak close to 28 ppm corresponds to oriented lipids, whereas the broad distribution and the peak around −15 ppm come from unoriented lipids. The ^15^N-NMR powder spectrum at the top encompasses the entire range of chemical shifts: an oriented signal as 65 ppm (black dotted line) indicates that a peptide lies flat on the membrane surface; higher ppm values indicate that the helix is slightly tilted in the membrane, while 180 ppm indicates an upright transmembrane alignment (not seen here).

While OCD provides a rough picture of the peptide alignment, the helix tilt angle can be measured rather precisely by solid-state NMR using the same type of oriented membrane sample. The chemical shift of a ^15^N-label in the peptide backbone reveals the angle between the helix axis and the bilayer normal: a ^15^N-NMR peak close to 70 ppm indicates a flat orientation of the peptide on the membrane surface with the helix axis perpendicular to the membrane normal. A peak at around 180 ppm indicates that the helix has a transmembrane orientation, while peaks between 70 and 180 ppm represent peptides with accordingly tilted orientations [21]. If the peptides are not helical, then the orientation cannot be determined from these ^15^N-NMR spectra (which is why CD/OCD need to be performed first), but other information may be available. Oriented NMR samples were prepared at a P/L of 1/100 in DMPC/DMPG (7/3), as for CD/OCD. The ^15^N-NMR spectra of all latarcins are summarized in Figure 7, along with the corresponding ^31^P-NMR spectra of the phospholipid component. The ^31^P-NMR spectra are a good indicator of (i) the quality of the membrane sample and can reveal (ii) any perturbing influence of the peptide on the lipid bilayer. As seen in Figure 7, most of the peptides are accompanied by a high proportion of well-oriented lipids. Only Ltc6a and Ltc7 display a large orientational spread of disordered lipids, suggesting that these long peptides can disturb the lamellar bilayer considerably. This conclusion is supported by the vesicle leakage data above. The ^31^P-NMR spectrum of Ltc2a shows a distinct second peak around 20 ppm, which is indicative of phase segregation of DMPC and DMPG in the presence of the peptide, suggesting that the anionic DMPG tends to cluster around this peptide with the highest charge density per residue.

Except for Ltc6a, all other peptides were found to be helical in the oriented membrane samples according to OCD; therefore, we can now use the ^15^N-NMR spectra to determine their orientation in the membrane more accurately. Ltc1, Ltc4a, Ltc5, and Ltc7 give ^15^N-NMR signals around 70 ppm, indicating that these helices lie flat on the membrane surface. Ltc2a, with a chemical shift of 131 ppm, on the other hand, is distinctly tilted in the membrane, as was also observed in the OCD experiments. According to our previous analyses of similar helical peptides, this chemical shift corresponds to a helix tilt angle of around 30–40° away from the flat surface orientation [22,23,24]. Ltc3a, with a chemical shift of 93 ppm, is tilted only slightly by 10–20°. Ltc6a has a relatively broad ^15^N-NMR signal at 82 ppm, and the ^31^P-NMR spectrum shows that this peptide strongly perturbs the lipid bilayer. Given that OCD had shown distinct signs of aggregation into β-sheets, we can conclude that Ltc6a binds to the membrane and perturbs the lipid bilayer, but it is not possible to determine the molecular alignment of Ltc6a when it has most likely formed aggregates. 

## 3. Discussion

In this work, we present a biophysical characterization of the full set of seven latarcin peptides in membranes, using CD, OCD, NMR, and vesicle leakage assay. Furthermore, we correlate these results with the antimicrobial and hemolytic activities of latarcins. These data are summarized in Table 2. All latarcins used in this study were synthesized with a backbone ^15^N-label and purified before use. As described in the introduction, we divided the seven peptides into four groups based on their (genetically unrelated) sequences and properties. Here, we evaluate and discuss our results.

Similar to most membrane-active peptides, all latarcins remained essentially unfolded in aqueous phosphate buffer (Supplementary Figure S2). However, in the presence of vesicles composed of DMPC/DMPG (7/3) at a P/L of 1/50, all of them were found to fold into the expected amphipathic α-helical structures, as illustrated in Figure 1. The CD line shapes (Figure 2) were deconvoluted to estimate the secondary structure contributions (Supplementary Table S1) using the CDSSTR, CONTIN-LL, and SELCON-3 algorithms provided on the DICHROWEB online server as described previously [17]. In group A, Ltc1 and Ltc2a show nearly identical spectral features with bands centered around 208 nm and 224 nm except for the positive band, which shows some blueshift for Ltc1 towards lower wavelength with reduced intensity, indicating that some unordered part may be present in this peptide. This result is also reflected in the helix content, which is significantly reduced for Ltc1 (66%) compared to Ltc2a (81%). Figure 1 shows that the two peptides have a similar hydrophobic sector, but Ltc2a has more distinct segregation between the polar and nonpolar residues, which favors better membrane binding and folding. In Ltc1, on the other hand, two charges (Lys10 and Lys21) are placed within the hydrophobic patch, as seen in the helical mesh representation. Group B peptides also have almost identical CD spectra and show the highest helix content of 87% to 94% (Table 2). These two peptides have the most distinct segregation of polar and nonpolar residues in the folded form and are therefore expected to build ideal α-helices, as shown in Figure 1. Group C (Ltc5) also shows similar hydrophobic and hydrophilic sectors, and this peptide is also expected to form an α-helix where most residues remain in the folded form. Despite the somewhat unusual line shape with a positive band shifted to the long wavelength and the rather intense negative band centered around 225 nm (in comparison to the band at 210 nm), Ltc5 indeed shows very high helicity (85%). The peculiar shift of the long-wavelength band can be attributed to the presence of a large number of aromatic residues (Table 1) in Ltc5. The helical mesh presented in Figure 1 shows that the hydrophobic sector is dominated by the presence of these aromatic residues. In peptides containing a high proportion of aromatic residues, such shifts of the long-wavelength band are well documented [25,26]. Group D peptides show rather large differences in their CD line shapes. Ltc6a has the lowest helicity (65%), whereas Ltc7 shows a pronounced line shape with a high helicity of 89% (Table 2). Given the nearly identical length and number of hydrophobic residues, this appears to be a surprisingly large difference in the folded fraction for these two peptides. In comparison to Ltc6a, Ltc7 has a larger and better separation between hydrophobic and hydrophilic sectors. Ltc6a actually carries several bulky hydrophobic residues (Tyr15, Met18, and Leu33) in its polar sector, unlike Ltc7, where Ala31 may be regarded as a rather inconspicuous side chain. These residues may cause some unfolding or perturb the binding of Ltc6a, resulting in an overall lower helicity. To conclude, the CD experiments, performed under identical conditions, were important because: (a) they show for the first time that all latarcins are highly helical in the presence of a lipid membrane, and (b) the evidently helical state allows a subsequent determination of the helix tilt angle using oriented circular dichroism and solid-state NMR techniques. 

The antimicrobial and hemolytic activities of latarcins have been reported previously [10]. In this work, we confirmed the antimicrobial action of latarcins on two Gram-positive and two Gram-negative bacterial strains. These results are presented in Figure 3, together with BP100, a well-known multifunctional antimicrobial peptide [27] that was used as a positive control. These data support the classification of latarcins into the suggested four groups. Ltc1 and Ltc2a, belonging to Group A, have the highest antimicrobial activity and closely resemble Ltc5 from Group C. This result may be expected due to the high charge per residue of these peptides (Table 1). Here, the MIC lies universally below 4 µg/mL irrespective of the bacterial strain, demonstrating their broad spectrum and rather indiscriminatory mechanism of bacterial killing. Next, only marginally less active are the Group B peptides comprising of Ltc3a and Ltc4a, where antimicrobial activity is observed in the range of 8–32 µg/mL. Similar to Groups A and C, Group B is also universally active on all strains. The peptides in Group D are less active. Here, Ltc6a inhibits the growth of the Gram-positive strains only, whereas Ltc7 does not inhibit bacterial growth up to a peptide concentration of 256 µg/mL. Although Ltc7 was found to be ineffective against the selected strains that have been tested here, it is conceivable that it may be active against other bacterial strains found in the habitat of the spider *L. tarabaevi* or that it may have some activity against other microorganisms or fungi. 

Hemolysis assays were performed on human erythrocytes, in contrast to earlier assays performed on rabbit erythrocytes in the literature [7]. Figure 4 shows that the hemolytic action of latarcins varies widely. The Group A peptides (Ltc1 and Ltc2a) have the highest hemolytic activity, with Ltc2a being the most hemolytic of all. They are followed by the Group C peptide (Ltc5), which is less hemolytic than Group A peptides. Group B (Ltc3a and Ltc4a) shows rather low hemolysis, which is noteworthy given their high antimicrobial action. Finally, Group D (Ltc6a and Ltc7) showed no or marginal hemolysis. The order of the hemolytic activity of the seven latarcins thus follows the same order as of the antimicrobial activity (Table 2). This finding suggests that the peptides have an indiscriminatory effect on the lipid membranes of bacteria or erythrocytes. Apparently, those peptides that are able to kill bacteria are also able to induce hemolysis of human erythrocytes, despite their fundamentally different lipid compositions, electrostatic properties, and transmembrane potentials. 

We may, at this point, speculate about two hypothetical conclusions regarding the biological membrane activity of the latarcin peptides. The first observation is that latarcins do not seem to be able to discriminate between different target membranes, given the same relative activities against different types of biomembranes, i.e., of prokaryotic and eukaryotic cells. This unspecific target selection of latarcins appears quite plausible, considering the venom’s purpose to disinfect the venom gland, paralyze and sterilize the prey, and inflict pain and deter predators. The second hypothesis is that the peptides themselves have encoded distinctly different degrees of activity and may therefore display functional specificity toward lipid membranes in general. This means, for example, that Group D peptides (Ltc6a and Ltc7) do not show much activity in bursting (bacterial or erythrocyte) membranes but may display some rather different kinds of membrane activity, such as cell penetration or others (which have NOT been tested here). At the same time, peptides in Group A were found to be highly active against bacteria and erythrocytes, but they may not show a good ability for more subtle membrane interactions. 

One way to test these hypotheses is to study the effects of the peptides against well-defined model membrane systems using a fluorescence leakage assay, which shows whether the peptides can directly induce rapid membrane permeabilization. Here, we used POPC/POPG (1/1) bilayers with a high proportion of anionic lipids. We found that our results support the concept of functional specificity. Ltc2a is extremely effective at inducing leakage in POPC/POPG vesicles, making this peptide an overall “membrane buster” that has an indiscriminatory response to bacterial membranes, eukaryotic membranes, and vesicles composed of synthetic lipids. The other Group A peptide, Ltc1, shows very low fluorescent leakage despite having similar properties as Ltc2a, and the same is true for Group C (Ltc5). It may have been expected that the high positive charge/residue ratio of these two peptides should provide sufficient electrostatic attraction to anionic vesicles and thus induce a high extent of leakage. Instead, we found that they induced the least leakage in vesicles made from POPC/POPG. From the CD and NMR results, it is clear that these peptides bind well but do not aggregate or perturb the bilayer, yet they are antimicrobial (Table 2). Taken together, these results suggest that the two peptides, Ltc1 and Ltc5, might have intracellular targets in bacteria and can penetrate the lipid bilayer without much physical damage. The two Group B peptides (Ltc3a and Ltc4a) show a relatively low extent of leakage (20%), comparable to their low hemolysis, with moderate antimicrobial activity. Group D peptides (Ltc6a and Ltc7), on the other hand, show either low or no antimicrobial (or hemolytic) activity but induce considerable vesicle leakage of 50% (Table 2). This means that they show specific action against the PC/PG model membranes but not against bacteria and erythrocytes. This finding suggests that the last two peptides, which showed no activity in our biological assays, may have other activities that are relevant for the spider.

Another possibility that we investigated here was the question of whether there could be any synergistic effects between the peptides. It is conceivable that some peptides do not show any impressive activity per se but turn out to be highly active in combination with another peptide partner. Such synergy is readily tested in a leakage assay because much smaller amounts of peptides are needed compared to antimicrobial and hemolytic experiments, and many samples can be prepared and measured effectively. All pairwise combinations of the seven latarcins were tested, but no synergy was found for any pair.

To obtain some molecular impressions of the underlying peptide-lipid interactions, the membrane alignment of the latarcins was examined using two complementary techniques, namely OCD and solid-state ^15^N-NMR. Both of these methods use identical sample preparation, where lipids are oriented on glass surfaces and well hydrated but without any excess water. Under these conditions, the peptides remain fully bound to the membrane and do not float away into regions of bulk water. The orientation of the α-helical peptides can be determined using these two methods, as seen in Figure 6 and Figure 7. OCD clearly shows that within Group A, the helix of Ltc1 is found to lie flat on the membrane surface. Ltc2a, on the other hand, is the most tilted peptide of the entire series. Interestingly, Ltc2a also has the highest membrane permeabilizing activity and the least selective effect on bilayers of different compositions. Group B peptides (Ltc3a and Ltc4a) show similar spectral profiles, indicating that both are surface-bound or very slightly tilted in the membrane. Ltc5 (Group C) and Ltc 7 in Group D were also found to lay flat on the membrane surface in the OCD experiments. Our results clearly indicate that when peptides bind to the membrane, they may not fold completely, even though they seemed to fold from the CD line shapes in TFE or in the presence of bulk water; thus, OCD is a unique way of determining the fold change of the peptides in the membranous environment. Such structural transitions have been observed previously, where otherwise helical peptides tend to unfold to aggregate as β-sheets in the 2D plane of the membrane [28,29]. In fact, Ltc6a loses its helical fold and aggregates in the oriented membrane samples prepared for OCD and ^15^N-NMR analysis; therefore, one cannot determine its alignment within the membrane.

The solid-state ^15^N-NMR results (Figure 7) nicely confirm the peptide orientation observed by OCD (Table 2). In these experiments, Ltc2a was found to be the most tilted helix in the membrane, with a signal around 130 ppm. Ltc3a is tilted slightly in the membrane, with a peak around 93 ppm, in line with our OCD results. All other latarcins show a ^15^N-NMR signal around 64–68 ppm, indicating that these helices are surface-bound. (Additionally, Ltc5 shows a signal clearly indicative of a surface-bound state, which confirms our interpretation of the OCD data above, which had been challenged by the presence of numerous aromatic residues.)

The ^31^P-NMR data show that most peptides do not perturb the lamellar bilayer order. The Group D peptides are an exception in producing a high mosaic spread (i.e., orientational disorder) of the lipids, and indeed they also show high vesicle leakage. Perhaps the most striking observation here is the fact that Ltc2a is the only peptide to induce lipid segregation: besides the usual signal at 26 ppm, a second ^31^P-NMR peak is seen to have emerged around 21 ppm in Figure 7. Such additional signal is sometimes observed in ^31^P-NMR of cationic amphiphilic peptides in a DMPC/DMPG matrix [30,31], especially in cases where lipid clustering has been demonstrated [32]. We, therefore, attribute the ^31^P-NMR signature of Ltc2a to its ability to induce lipid segregation due to its high charge. Furthermore, a significant upfield shift of a ^31^P-NMR signal would be expected if the lipids are motionally averaged by diffusing through a highly curved environment. The latter effect would indeed be plausible if Ltc2a were to cause a dimple in the bilayer due to its high tilt angle of 30–40° away from the membrane surface according to ^15^N-NMR. 

Compared to the other latarcins, it is likely that the very high charge density in Ltc2a (11 Lys amongst 26 amino acids, see Table 1) and its pronounced amphiphilicity (11 hydrophobic residues) contribute to its particularly disruptive membrane interactions. The most unusual feature of Ltc2a, in our opinion, is the strip of hydrophobic amino acids that does not run in parallel along but rather diagonally around the helix axis, as seen in the helical mesh representation of Figure 1. This amphiphilic profile is closely reminiscent of a coiled-coil motif—a pattern that is typically known to drive the assembly of two (or more) α-helices in an aqueous solution. In the case of Ltc2a, however, we suggest that this particular structural element is instead critically important in modulating its preferred mode of membrane binding. Given the 180° angle by which the hydrophobic strip is wrapped around the helix, the fully folded Ltc2a molecule is expected to be quite unable to bind straight onto the flat membrane surface, unlike most other known amphiphilic peptides. Instead, the Ltc2a helix will be forced to insert more steeply into and possibly across the lipid bilayer in a slanted manner in order to optimize its hydrophobic contacts with the lipids, as was indeed observed here by OCD and NMR (Figure 6 and Figure 7). This unusual kind of coiled-coil amphiphilicity is described and interpreted here for the first time with regard to a membrane-active peptide. It should be noted that Ltc1 possesses a similar coiled-coil pattern at first sight (see Figure 1), but its hydrophobic strip is interrupted by two cationic residues (Lys10, Arg15). Therefore, it is much easier for Ltc1 to adjust to the flat membrane surface by kinking. Indeed, the helicity of Ltc1 is found to be lower than for Ltc2a in the membrane-bound state, according to CD (Figure 2). The other members of the latarcin family display no coiled-coil features, as their amphiphilic profiles run parallel to the helix axis in all cases. With its peculiar and well-defined coiled-coil motif and its resultant vigorous membrane interactions, Ltc2a stands out distinctly from Ltc1 and the other latarcins and also in all of the functional assays that were studied here. Ltc2a is found to be the most aggressive and least selective membrane-permeabilizing agent, with an extremely high antimicrobial, hemolytic, and leakage-inducing activity. 

In summary, our results show that the amphiphilic latarcin helices lie on the membrane surface or can tilt slightly into the lipid bilayer. It is tempting to speculate that, mechanistically, the entire group of seven peptides covers a wide spectrum of activities and specificities. Some act via lipid clustering and massive permeabilization (Ltc2a) or by disordering the lamellar bilayer (Ltc6a, Ltc7), while others might be able to translocate non-perturbingly towards intracellular targets (Ltc1, Ltc5). Some are highly antibiotic but show low hemolytic effects (Ltc3a, Ltc4a, Ltc5a), while others destroy membranes indiscriminately (Ltc2a), and yet, others are inefficient antibiotics but permeabilize lipid bilayers (Ltc6a, Ltc7). Given the absence of any pairwise synergy, these diverse features highlight the multiple purposes of spider venom. 

## 4. Materials and Methods

### 4.1. Materials

^15^N-labelled amino acids were purchased from Cambridge Isotope Laboratories (Andover, MA, USA) and were Fmoc-protected using Fmoc-Cl as described previously [33]. The lipids 1,2-dimyristoyl-*sn*-glycero-3-phosphatidylcholine (DMPC), 1,2-dimyristoyl-*sn*-glycero-3-phosphatidylglycerol (DMPG), 1-palmitoyl-2-oleoyl-*sn*-glycero-3-phosphatidylcholine (POPC), and 1-palmitoyl-2-oleoyl-*sn*-glycero-3-phosphatidylglycerol (POPG), were purchased from NOF Europe (Grobbendonk, Belgium). The fluorescent lipid 1,2-dioleoyl-*sn*-glycero-3-phosphoethanolamine-N-(lissamine rhodamine B sulfonyl) (Rhod-PE) were obtained from Avanti Polar Lipids. The fluorescent probes 8-amino-naphtalene-1,3,6-trisulfonic acid sodium salt (ANTS) and p-xylene-bis(pyridinium)bromide (DPX) were obtained from Invitrogen-Molecular Probes (Karlsruhe, Germany).

### 4.2. Peptide Synthesis

Latarcins were synthesized using Fmoc solid-phase peptide synthesis protocols as previously described [32,34,35]. Briefly, as listed in Table 1, Ltc1, Ltc2a, Ltc6a, and Ltc7 were synthesized as C-terminal acids using Fmoc-Glu(OtBu)-Wang resin, Fmoc-His(Trt)-Wang resin, Fmoc-Leu-Wang-LL-resin, and Fmoc-Ser(OtBu)-Wang-LL-resin, respectively. Ltc3aa, Ltc4a, and Ltc5 were synthesized as C-terminal amides using Rink Amide MBHA resin. Automated peptide synthesis was performed on a Syro II multiple peptide synthesizer (Syro II; MultiSyntech, Witten, Germany) on a 100 µmol scale except for Ltc6a and Ltc7 for which an equivalent of 50 µM low load (LL) resin was used. Double coupling was performed using 100 µM chemistry with 4 equivalent excesses of amino acids using HOBt/HBTU in the presence of DIPEA. For solid-state NMR experiments, each latarcin was labeled at the backbone nitrogen at the underlined position (Table 1) using Fmoc-^15^N-Leu, Fmoc-^15^N-Ile, or Fmoc-^15^N-Phe. These amino acids were coupled manually using 2 equivalent excesses of isotope-labeled amino acids using the same method as described above except that the coupling was extended for overnight to ensure complete coupling. Crude peptides were cleaved using previously described methods [32,34,35] and purified using C18 semi-preparative high-performance liquid chromatography (HPLC) methods with water/acetonitrile gradients containing 5 mM HCl as described previously [35,36]. Gradients were individually optimized for each peptide to obtain high purity. The identity and purity (>95%) of the peptides was confirmed using analytical HPLC (Agilent Technologies, Waldbronn, Germany) coupled to a mass spectrometer (q-TOF, Bruker, Bremen, Germany). 

### 4.3. Vesicle Leakage Assay

For the leakage experiments, the buffer in which the vesicles were prepared contained the fluorophore ANTS (12.5 mM), the quencher DPX (45 mM), 50 mM NaCl, and 10 mM HEPES (pH 7.5). Liposomes were prepared by co-dissolving POPC/POPG 1/1 (mol/mol) lipids in CHCl_3_/MeOH (3/1 *v*/*v*), together with 10^−2^ mol% Rhod-PE, by which the lipid loss during vesicle preparation (extrusion and gel filtration, see below) could be quantified. The peptide-to-lipid ratio (P/L) is given in mol/mol. The lipid mixture was dried under N_2_(g) and dried under vacuum for 2–4 h. The obtained thin film was then re-suspended in the buffer, which contained the fluorophore and the quencher, by vigorous vortexing, followed by 10 freeze–thaw cycles. Large unilamellar vesicles (LUV) were obtained by 41-fold extrusion (Avanti Mini Extruder; Avanti Polar Lipids, Alabaster, AL, USA) of the liposomes through a Nuclepore polycarbonate membrane (pore size 100 nm, Whatman-GE Healthcare Europe, Freiburg, Germany) at a temperature 20 °C above the lipid phase transition. Unencapsulated dye was removed by gel filtration using spin columns filled with Sephacryl 100-HR (Sigma-Aldrich, Taufkirchen, Germany) and equilibrated with an elution buffer (150 mM NaCl, 10 mM HEPES, pH 7.5), which balances the internal vesicle osmolarity.

The leakage of entrapped ANTS was monitored by fluorescence dequenching of ANTS [18]. Fluorescence measurements were performed in a thermostated cuvette with constant stirring at 30 °C in the elution buffer used for gel filtration on a FluoroMax2 spectrofluorimeter (HORIBA Jobin Yvon, Unterhaching, Germany) by setting the ANTS excitation wavelength to 355 nm and its emission wavelength to 510 nm. Peptide stock solutions in water were prepared, and the peptide concentration was calculated from the weighed amount. The vesicles (100 µM lipid final concentration) were added to the cuvette containing the peptide at a concentration according to the P/L ratio to be tested. The fluorescence signal was followed for 10 minutes, and after that time, Triton X-100 was added to completely disrupt the vesicles. The level of 0% leakage corresponded to the fluorescence of the vesicles immediately after their addition, while 100% leakage was the fluorescence value obtained after the addition of Triton X-100. 

The synergy of the peptides towards vesicle leakage was investigated by pair-wise combinations of latarcins. The leakage was measured for each peptide alone at P/L = 1/100 and for peptide combinations at P/P/L = 1/1/100. The synergy factor, SF, is calculated according to:

SF = L(P1 + P2)/[L(P1) + L(P2)].(1)

Here, L(P1) is the leakage of peptide 1 alone, L(P2) is the leakage of peptide 2 alone, and L(P1 + P2) is the leakage for the mixture of the peptides. An additive effect gives a synergy factor of 1, whereas a synergy factor of 2 or more would indicate synergy. Peptides were mixed in the buffer before vesicles were added. The experiments were repeated at least three times using different batches of vesicles and peptide solution. The SF was determined separately for each batch of samples, and then the individual SF values were averaged.

### 4.4. Circular Dichroism Spectroscopy 

Small unilamellar vesicles (SUVs) for the CD measurements were prepared as follows. Weighed amounts of the lipid powders (DMPC, DMPG) were dissolved in chloroform/methanol 1/1 (*v*/*v*) to obtain 5 mg/mL stock solutions. For obtaining mixed DMPC/DMPG liposomes (7/3 molar ratio), the appropriate aliquots of these stock solutions were pipetted in a glass vial and thoroughly vortexed. The organic solvents were removed under a gentle stream of nitrogen gas, followed by 3 h of vacuum pumping in an exsiccator at a reduced pressure of 3-4 mbars to remove solvent residuals from the lipid film that had formed in the vial. The lipid film was dispersed by the addition of 10 mM phosphate buffer (PB) pH 7.0 and homogenized by vigorously vortexing for 10 × 1 min and 10 freeze–thaw cycles. SUVs were formed by sonication of the obtained multilamellar vesicles for 16 min in a strong ultrasonic bath (UTR 200, Hielscher, Germany). The temperature of the water in the ultrasonic bath was adjusted to 35 °C by circulating it through a thermostat to avoid overheating the samples. A weighed amount of the corresponding peptide was dissolved in water resulting in stock solutions with a peptide concentration of 0.5 mg/mL. For the final CD samples, each peptide stock solution was diluted to 29.3 μM and the corresponding lipid dispersion to 1.47 mM in 10 mM PB, resulting in a peptide-to-lipid (P/L) ratio of 1/50. 

CD spectra of the latarcins in membrane-mimicking DMPG / DMPC 7/3 liposomes were recorded on a J-815 spectropolarimeter (JASCO, Groß-Umstadt, Germany). The samples were measured in a 1-millimeter path length quartz glass cell (Suprasil, Hellma Müllheim, Germany) between 260 and 185 nm at 0.5 nm intervals. Spectra of the liposome samples were recorded at 30 °C, i.e., well above the phase transition temperature of the lipids, using a water-thermostated rectangular cell holder. Three repeat scans at a scan rate of 10 nm min^−1^, 8 s response time, and 1 nm bandwidth were averaged for each sample, and the baseline spectrum of the respective peptide-free sample was subtracted from the sample spectrum. CD spectra were smoothened by a Savitzky-Golay filter (convolution width of 15 data points), which is implemented in the Jasco Spectra Analysis software. The molar concentration of each latarcin peptide stock solution was determined based on the weight of the added peptide and the volume of the solution. The peptide concentration in the final liposome CD samples was calculated from the respective dilution factors and used for conversion to mean residue ellipticity units.

### 4.5. Oriented Circular Dichroism Spectroscopy 

To prepare oriented CD (OCD) samples, latarcins depending on their solubility were dissolved either in pure MeOH or CHCl_3_/MeOH 1/1 (*v*/*v*) and thoroughly mixed with appropriate aliquots of DMPC/DMPG solutions (7/3 molar ratio) in the same solvent. Each co-dissolved mixture with a peptide-to-lipid molar ratio of 1/100 was deposited on a quartz glass plate using a gas-tight syringe. To achieve a spot size of 12.5 mm on the 20 mm diameter plates (Suprasil QS, Hellma Optik GmbH, Jena, Germany), a volume of ~55–60 µL was used. The organic solvents were evaporated in a gentle stream of air until the sample appeared dry. The amounts of deposited lipid and peptide on the plate were always 0.29 μmole and 2.9 nmole, respectively. The peptide-lipid-film was further dried under reduced pressure for at least 3 h. Afterwards, each plate with the dried sample was fixed and sealed by a polymer O-ring in a cylindrical sample holder. The plate with the sample and a second window plate without the sample formed a compartment for controlled hydration. The holder was assembled in a custom-built hydration cell. In the inner compartment of the sample holder, the peptide-lipid film was hydrated for about 15 h at 30 °C and 97% relative humidity via the gas phase by a saturated K_2_SO_4_ solution contained in an adjacent cavity of the hydration cell. During hydration, the lipids spontaneously align as lipid bilayers that are macroscopically oriented perpendicular to the glass surface. For the measurement, the holder was transferred to an OCD sample cell that had been developed and built in-house [20]. It is computer-controlled and can be integrated into a Jasco spectropolarimeter as an accessory. The optical path runs along the cylindrical axis of the cell, i.e., normal to the quartz glass plate surface carrying the oriented lipid film. To reduce possible spectral artifacts caused by linear dichroism (LD) or linear birefringence (LB) due to imperfections in the sample, strain in the quartz glass or imperfect alignment of the windows, the OCD spectra were recorded in steps of 45° by rotation of the cell around the beam axis [19,20]. At each angle, two scans were recorded from 260–185 nm as an average, using a scan rate of 20 nm min^−1^, 4 s response time, and 1 nm bandwidth. The same parameters were chosen for the baseline of the respective peptide-free sample. The eight rotational spectra were subsequently averaged, and the baseline spectrum of lipid bilayers without peptide was subtracted. For a better comparison of spectral features, the OCD spectra of the different latarcins were normalized to an arbitrary ellipticity value of −10 mdeg at the corresponding long-wavelength negative peak maximum and the spectra in Figure 6 are shown in arbitrary units (a.u.).

### 4.6. Solid-State ^15^N-NMR

Macroscopically oriented NMR samples were prepared by co-dissolving appropriate amounts of peptides and lipids (in 300 μL methanol, 100 μL CHCl_3_, and 10–20 μL Milli-Q water) and spreading them onto 23 thin glass plates of dimensions 9 mm × 7.5 mm × 0.08 mm (Marienfeld Laboratory Glassware, Lauda-Königshofen, Germany). The peptide-to-lipid ratio (P/L) is given in mol/mol. The plates were dried in air for 1 h, followed by drying under vacuum overnight. The plates were stacked and placed into a hydration chamber with 96% relative humidity at 48 °C for 18–24 h before wrapping the stack in parafilm and plastic foil for the NMR measurements. All solid-state NMR measurements were carried out on a Bruker Avance 500 MHz spectrometer (Bruker Biospin, Karlsruhe, Germany) at 308 K, as previously reported [23,24,37,38]. The oriented membrane samples were placed in the flat-coil probe such that the lipid bilayer normal was aligned parallel to the magnetic field. ^31^P-NMR was used to check the quality of the lipid orientation in the samples, using a Hahn echo sequence with phase cycling [39]. The 90° pulse length was 3.9 µs, the echo time was 30 µs, and 17 kHz ^1^H SPINAL64 decoupling was used. ^1^H-^15^N cross-polarization experiments using a CP-MOIST pulse sequence [40] were performed using a double-tuned probe with a low-E flat-coil resonator (3 mm × 9 mm cross-section), employing a ^1^H and ^15^N radiofrequency field strength of 65 kHz during the cross-polarization and 25 kHz ^1^H SPINAL16 [41] decoupling during acquisition. A mixing time of 500 μs was used, and 10,000 to 30,000 scans were accumulated. The acquisition time was 10 ms, and the recycle time was 3 s. The ^15^N chemical shift was referenced using the ^1^H-NMR signal of water in the sample. 

### 4.7. Minimum Inhibitory Concentration

The minimum inhibitory concentration (MIC) was determined as previously reported [35,42]. For each latarcin, MIC was determined against two Gram-positive strains, namely *Bacillus subtilis* subsp. *spizizenii* (DSM 347) and *Staphylococcus xylosus* (DSM 20267), and two Gram-negative strains: *Escherichia coli* (DSM 1116) and *Acinetobacter* sp. (DSM 586). Bacterial cultures were grown in Müller-Hinton medium (MHM) as described previously [42], and peptides were dissolved in sterile water containing 10% DMSO. The stock solution of peptides with 1024 µg/mL was prepared such that serial dilutions of peptides (from 256 µg/mL to 2 µg/mL) in MHM were prepared in 96-well plates with the penultimate column without peptide to act as positive control (no peptide) and the final column as negative control (no bacteria). Bacterial stock suspension was made with an OD of 0.2 units at 550 nm. This stock solution was further diluted (1:1000 for Gram-negative bacteria and 1:100 for Gram-positive bacteria), and subsequently, 50 µL of the resulting bacterial suspension was added to the wells except the final well to give a final concentration of 10^6^ colony-forming units per mL. Afterwards, the 96-well plates were incubated at 37 °C for 20 h, and cellular respiration was detected by adding 20 µL of resazurin solution (200 µg/mL) for an additional 2 h at the same temperature. The MIC value was determined by detecting a distinct color change from blue to pink at the lowest concentration of the peptide, which successfully inhibited bacterial growth.

### 4.8. Hemolysis Assay

The influence of latarcins on human erythrocytes was determined by using a hemolysis assay modified from previously published methods [35]. Blood was obtained from the blood bank of the local hospital in Karlsruhe and stabilized with citrate phosphate dextrose buffer. Erythrocyte suspension (5 mL) was taken up in Tris buffer (45 mL, 172 mM, pH 7.6) at room temperature, gently mixed, and centrifuged (1500 rpm, 10 min). The supernatant solution was removed, and Tris wash was repeated twice with 45 mL as above. The washed erythrocytes (1 mL) were suspended in 9 mL Tris buffer to make the stock cell suspension, which was then further diluted to 0.25% (*v*/*v*) at 37 °C before use. Peptide stock solutions were prepared with 1024 µg/mL in Tris buffer containing 10% DMSO, and serial dilutions were prepared in the range of 512 µg/mL to 2 µg/mL as described previously [43]. For the positive control, Triton X-100 (200 µL of 0.2% *v*/*v*) was used, and as the negative control, Tris buffer (200 µL, 172 mM) was used. Erythrocyte stock cell suspension (200 µL) was added to each of the samples and incubated for 30 min at 37 °C with gentle shaking before determining the percentage of hemolysis as described previously [35,43]. 

## 5. Conclusions

In summary, we characterized the full set of latarcins in their membrane-bound state using CD, OCD, and NMR experiments. This work adds new information on the secondary state of peptides in the membrane, characterizes membrane alignment, and highlights peptides that show a high aggregation tendency in the membrane. Our results show that latarcins fold into α-helices upon membrane binding, as shown by solution CD experiments (Figure 2). OCD and NMR data show that most of the helical peptides are surface-bound or tilted in the membrane, which may further explain their mode of action (Figure 6 and Figure 7). The only exception is Ltc6a, which shows a low helix content in the presence of bulk water but tends to aggregate in the membrane. Our work demonstrates how a given family of peptides may act very differently depending on the target membrane (Figure 5). Ltc1, Ltc2a, Ltc3a, Ltc4a, and Ltc5 are important peptides with high antimicrobial activity (Figure 3). With the exception of Ltc2a, all show rather low hemolytic side effects or vesicle leakage and are therefore promising candidates for further optimization (Figure 4 and Figure 5). Furthermore, low leakage and low membrane perturbation, as seen by the solid-state NMR of most of the active latarcins, indicate that latarcins may have intracellular targets responsible for bacterial killing. Given the rise in antimicrobial infections, such naturally optimized peptides with fewer side effects may offer promising antibiotics in the future. 

## Figures and Tables

**Table 1:** Sequences and properties of the seven latarcin peptides.

Name	Sequence ^a^	L ^b^	Charged Residues ^c^	C/r ^d^	A. r ^e^	H. r ^f^	MW ^g^
Ltc1	**SMWSGMW** **RRK** **L** **KK** ** L ** **R** **NAL** **KKK** **L** **K** **G** **E** **-acid**	25	+11 − 2 = +9	0.36	2	9	3075
Ltc2a	**GLFG** **K** **LI** **KK** **FG** **RK** **AISYAV** **KK** **A** **R** **G** **KH-** **acid**	26	+11 − 1 = +10	0.38	3	11	2904
Ltc3a	**SW** **K** **SMA** **KK** ** L ** **K** **E** **YM** **E** **K** **L** **K** **Q** **R** **A-amide**	20	+8 − 2 = +6	0.30	2	8	2484
Ltc4a	**GL** **K** **D** **K** **F** **K** **SMG** **E** **K** ** L ** **K** **QYIQTW** **K** **A** **K** **F-amide**	24	+8 − 2 = +6	0.25	4	9	2903
Ltc5	**GFFG** **K** **M** **K** **E** **YF** **KK** **FGASF** **KRR** **FANL** **KKR** **L-amide**	28	+11 − 1 = +10	0.36	7	12	3431
Ltc6a	**QAFQTF** **K** **P** **D** **WN** **K** **I** **R** **Y** **D** **AM** **K** **MQTSLGQM** **KKR** **FNL-acid**	33	+8 − 3 = +5	0.15	5	13	4053
Ltc7	**G** **E** **TF** **D** **K** **L** **K** **E** **K** **L** **K** **TFYQ** **K** **LV** **E** **K** **A** **ED** ** L ** **K** **G** **D** **L** **K** **A** **K** **LS-acid**	34	+10 − 8 = +2	0.06	3	12	3944

^a^ Positive charges are marked in blue and negative charges in red, and the ^15^N-labeled position is underlined in each peptide. ^b^ Number of residues. ^c^ Positive charges, negative charges, and net charge. ^d^ Net charge per residue. ^e^ Number of aromatic residues. ^f^ Number of hydrophobic residues. ^g^ Molecular weight in g/mol.

**Table 2:** A summary of the biophysical and biological data of latarcins.

Peptide	% Helix (CD) ^a^	OCD ^b^	NMR ^b^	% Leakage ^c^	MIC ^d^	% Hemolysis ^e^
Ltc1	66	Surface	Surface	7	+++	100
Ltc2a	81	Tilted	Tilted	77	+++	36
Ltc3a	87	Slight tilt	Slight tilt	20	++	16
Ltc4a	94	Slight tilt	Slight tilt	22	++	12
Ltc5	85	Surface	Surface	4	+++	27
Ltc6a	65	Aggregated	Aggregated	46	+	3
Ltc7	89	Surface	Surface	54	−	2

^a^ CD in DMPC/DMPG (7/3), P/L = 1:50. ^b^ Orientational state of the helix in DMPC/DMPG (7/3), P/L = 1:100. ^c^ Vesicle leakage in POPC/POPG (1/1), P/L = 1:50. ^d^ Minimum inhibitory concentration (MIC) showing relative activity of latarcins. The antimicrobial activity is coded as: (−) inactive, (+) marginally active, (++) moderately active, and (+++) highly active. ^e^ Hemolytic activity determined at a peptide concentration of 256 µg/mL towards human red blood cells.

## Supplementary Materials

The following are available online at https://www.mdpi.com/article/10.3390/ijms221810156/s1, Figure S1: Analytical HPLC chromatograms of the purified latarcins, Figure S2: Circular dichroism spectra of latarcins in 10 mM phosphate buffer at a defined pH of 7 at 30 °C, Figure S3: Synergy of leakage for pairwise combinations of long latarcins, Table S1: Secondary structure fractions (in %) of Latarcins.
