# Supplementary material for: Membrane Interactions of Latarcins: Antimicrobial Peptides from Spider Venom
Source: Int J Mol Sci. 2021 Sep 21;22(18):10156. doi: 10.3390/ijms221810156 (PMC8470881; doi:10.3390/ijms221810156)
Supplement: Supplementary file 1 [file ijms-22-10156-s001.zip › ijms-1383135-supplementary.pdf]

Supporting Information

Membrane interactions of latarcins: antimicrobial peptides from spider venom

Parvesh Wadhwani[1], Saiguru Sekaran[2] , Erik Strandberg[1], Jochen Bürck[1], Archana Chugh[2] and Anne S. Ulrich[1,3,*]

1 Institute of Biological Interfaces (IBG-2), Karlsruhe Institute of Technology (KIT), POB 3640, 76021 Karlsruhe, Germany;
2 Kusuma School of Biological Sciences, Indian Institute of Technology Delhi, Hauz Khas, Delhi-110016, India;
3 Institute of Organic Chemistry, Karlsruhe Institute of Technology (KIT), Fritz-Haber-Weg 6, 76131 Karlsruhe, Germany;
* Correspondence: Anne.Ulrich@kit.edu

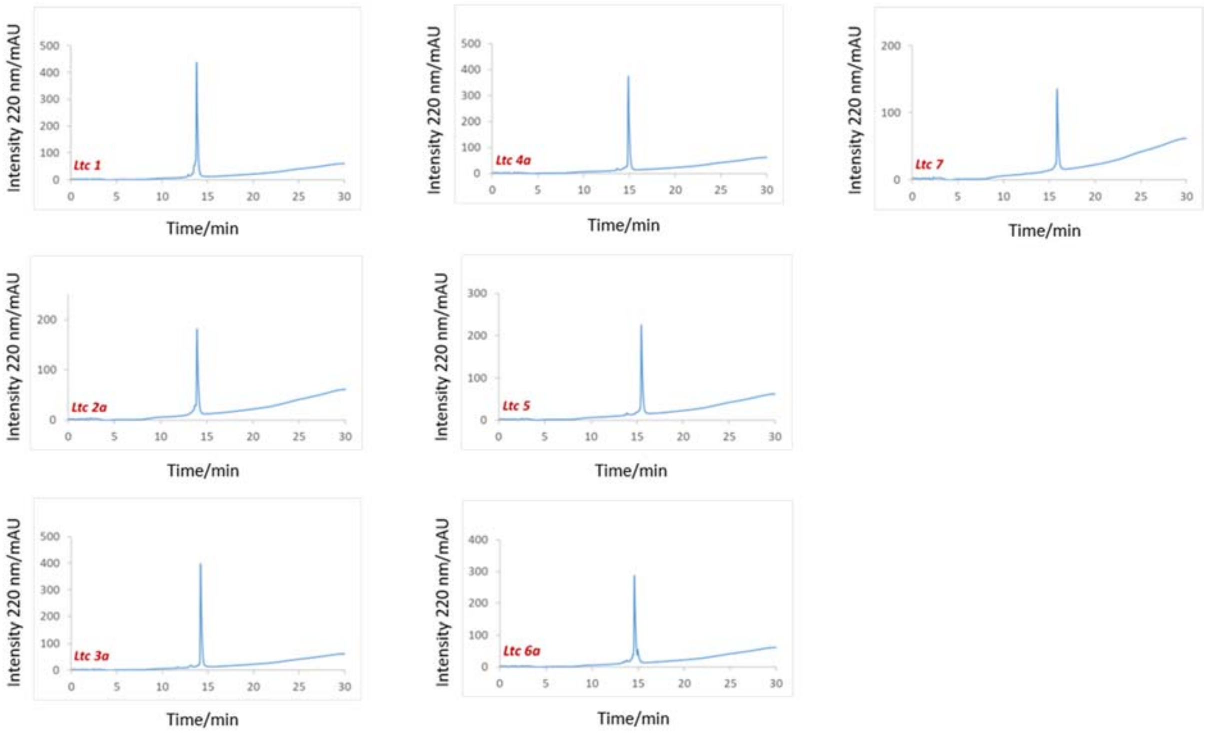

Figure S1: Analytical HPLC chromatograms of the purified latarcins.

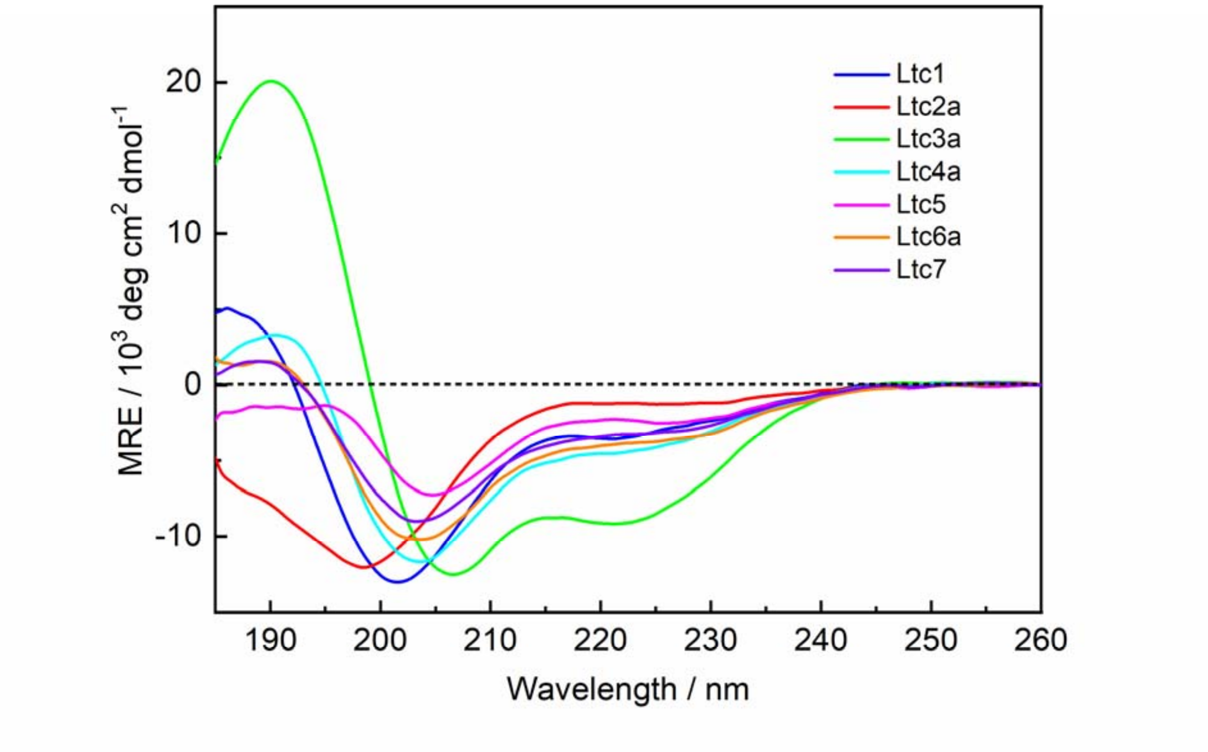

Figure S2. Circular dichroism spectra of latarcins in 10 mM phosphate buffer at a defined pH of 7 at 30°C. In comparison to the previously reported CD spectra of latarcins in pure water where all latarcins show an unordered structure, here, under high ionic strength and defined pH most latarcins show nascent helical conformation. Ltc3a shows a helical fold of about 30%. In addition, Ltc1, Ltc4a, Ltc6a, and Ltc7 also show a distinct shift of the negative band from 198 nm (as seen for Ltc2a) and have an emerging positive signal around 190 nm indicating the distinct change from the previously published spectra [1].

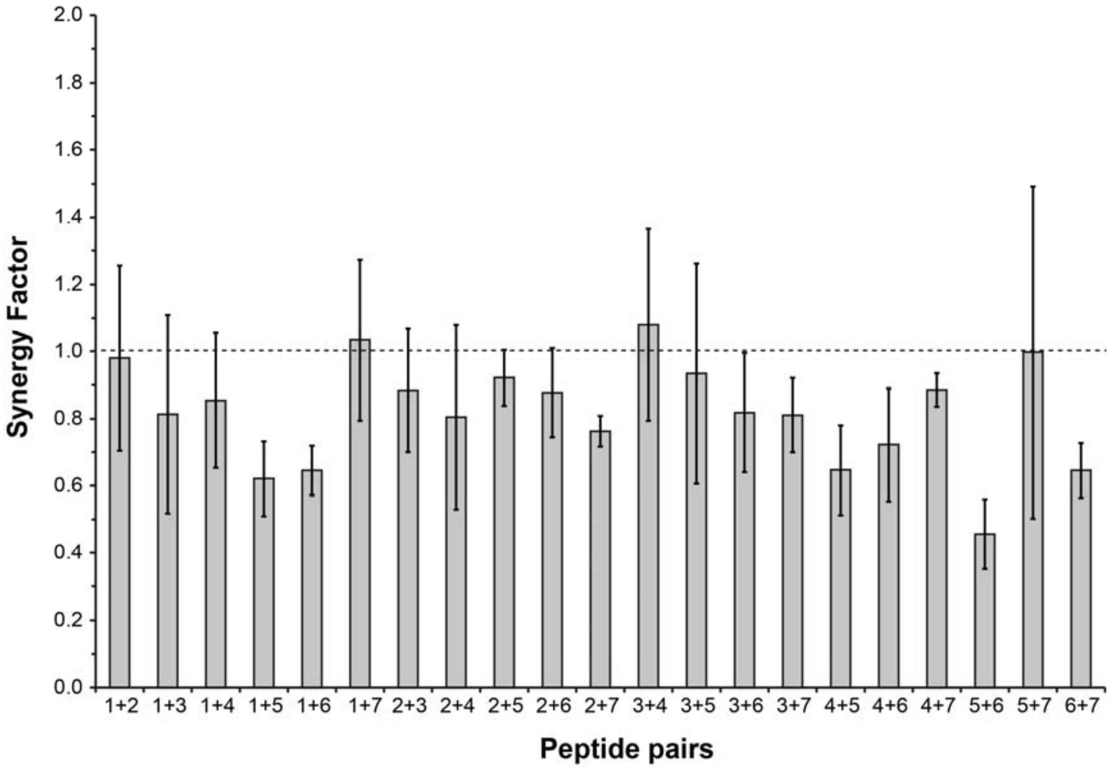

Figure S3. Synergy of leakage for pairwise combinations of latarcins. Leakage was measured for each peptide alone at P/L=1/100, and for peptide combinations at P/P/L=1/1/100. The synergy factor is calculated as the leakage of the peptide mixture divided by the sum of the individual peptide leakages. An additive effect gives a synergy factor of 1, whereas a synergy factor of 2 or more would indicate synergy. All peptide combinations show a synergy factor of 1 or below.

Table S1: Secondary structure fractions (in %)[a] of Latarcins in DMPC/DMPG (7:3) SUVs, evaluated from the CD spectra. Average values and the standard deviation for three different algorithms (CDSSTR, CONTIN-LL and the SELCON-3) are given.

Peptide	α-helix	β-strand	β-Turn	Unordered
Ltc1	66 ± 3	4 ± 1	10 ± 1	19 ± 3
Ltc2a	81 ± 4	2 ± 1	7 ± 1	11 ± 5
Ltc3a	87 ± 3	1 ± 2	6 ± 5	6 ± 4
Ltc4a	94 ± 3	1 ± 1	3 ± 1	3 ± 4
Ltc5	85 ± 5	6 ± 2	4 ± 5	5 ± 4
Ltc6a	65 ± 3	4 ± 1	12 ± 2	20 ± 2
Ltc7	89 ± 6	1 ± 1	2 ± 0	7 ± 6

[a] Due to rounding errors, the sum is not always exactly 100%. Secondary structure estimation from deconvolution of CD spectra was performed using the CDSSTR program with the implemented singular value decomposition (SVD) algorithm [2,3], using the CONTIN-LL program, which is based on the ridge regression algorithm [4,5], and using the SELCON-3 program, which incorporates the self-consistent method together with the SVD algorithm to assign protein secondary structure [6,7]. The three algorithms are provided by the DICHROWEB on-line server [8]. The secondary structure element percentages of each sample are given as the average values, and error ranges are given as the standard deviations of the individual calculated values obtained with the three algorithms. Individual values were not considered for the average if the sum of all structural elements fractions was <98% or >102%, or when the NMRSD was above the threshold value (0.1 for CONTIN-LL and CDSSTR, and 0.25 for SELCON-3) [9]. NRMSD is the unitless normalized root mean square deviation between experimental and back-calculated spectra.

References

[1] S.A. Kozlov, A.A. Vassilevski, A.V. Feofanov, A.Y. Surovoy, D.V. Karpunin, E.V Grishin, E.V., Latarcins, antimicrobial and cytolytic peptides from the venom of the spider Lachesana tarabaevi (Zodariidae) that exemplify biomolecular diversity. J. Biol. Chem. 2006, 281, 20983–20992.

[2] W.C. Johnson, Analyzing protein circular dichroism spectra for accurate secondary structures, Proteins, 35 (1999) 307-312.

[3] N. Sreerama, S.Y. Venyaminov, R.W. Woody, Estimation of protein secondary structure from circular dichroism spectra: inclusion of denatured proteins with native proteins in the analysis, Anal. Biochem., 287 (2000) 243-251.

[4] S.W. Provencher, J. Glockner, Estimation of globular protein secondary structure from circular dichroism, Biochemistry, 20 (1981) 33-37.

[5] I.H. van Stokkum, H.J. Spoelder, M. Bloemendal, R. van Grondelle, F.C. Groen, Estimation of protein secondary structure and error analysis from circular dichroism spectra, Anal. Biochem., 191 (1990) 110-118.

[6] N. Sreerama, S.Y. Venyaminov, R.W. Woody, Estimation of the number of a-helical and b-strand segments in proteins using circular dichroism spectroscopy, Protein Sci., 8 (1999) 370-380.

[7] N. Sreerama, R.W. Woody, A self-consistent method for the analysis of protein secondary structure from circular dichroism, Anal. Biochem., 209 (1993) 32-44.

[8] A. Lobley, L. Whitmore, B.A. Wallace, DICHROWEB: an interactive website for the analysis of protein secondary structure from circular dichroism spectra, Bioinformatics, 18 (2002) 211-212.

[9] L. Whitmore, B.A. Wallace, DICHROWEB, an online server for protein secondary structure analyses from circular dichroism spectroscopic data, Nucleic Acids Res., 32 (2004) W668-673.
